# Trafficking and effect of released DNA on cGAS-STING signaling pathway and cardiovascular disease

**DOI:** 10.3389/fimmu.2023.1287130

**Published:** 2023-12-13

**Authors:** Zimo Zhou, Changhan Ou-yang, Qingjie Chen, Zhanhong Ren, Xiying Guo, Min Lei, Chao Liu, Xiaosong Yang

**Affiliations:** ^1^ Hubei Key Laboratory of Diabetes and Angiopathy, Hubei University of Science and Technology, Xianning, China; ^2^ State Key Laboratory of Trauma, Burns and Combined Injury, College of Preventive Medicine, Army Medical University, Chongqing, China; ^3^ Xianning Medical College, Hubei University of Science and Technology, Xianning, China

**Keywords:** dsDNA, cGAS-STING, innate immune system, inflammation, cardiovascular disease

## Abstract

Evidence from clinical research and animal studies indicates that inflammation is an important factor in the occurrence and development of cardiovascular disease (CVD). Emerging evidence shows that nucleic acids serve as crucial pathogen-associated molecular patterns (PAMPs) or non-infectious damage-associated molecular patterns (DAMPs), are released and then recognized by pattern recognition receptors (PRRs), which activates immunological signaling pathways for host defense. Mechanistically, the released nucleic acids activate cyclic GMP-AMP synthase (cGAS) and its downstream receptor stimulator of interferon genes (STING) to promote type I interferons (IFNs) production, which play an important regulatory function during the initiation of an innate immune response to various diseases, including CVD. This pathway represents an essential defense regulatory mechanism in an organism’s innate immune system. In this review, we outline the overall profile of cGAS-STING signaling, summarize the latest findings on nucleic acid release and trafficking, and discuss their potential role in CVD. This review also sheds light on potential directions for future investigations on CVD.

## 1 Introduction

Overwhelming evidence indicates that cardiovascular diseases (CVD), one of the most common non-communicable diseases, has become a major cause of premature morbidity and mortality globally, account for approximately one third of all deaths worldwide (1). The CVD epidemic has devastating medical and economic consequences for governments and families. In the last century, our comprehension of CVD causation and the development of therapeutic medicine has seen considerable progress. According to current knowledge, therapeutic medicine includes, but are not limited to, those targeting factors that initiate inflammatory responses, involving innate immune system activation, cardiac hypertrophy, cardiomyocytes death, proliferation, and cardiac fibroblasts trans-differentiation. However, based on the actual that translating preclinical findings to clinical settings remains a major challenge, the detailed molecular mechanisms underlying cardiac pathophysiological conditions are yet to be elucidated. Additionally, substantial novel therapeutic targets are yet to be explored.

Several clinical and animal experimental studies found that CVD were usually accompanied by a chronic inflammatory response (2). In recent times, inflammatory pathology has garnered special attention owing to its function in the initiation and development of CVD, including heart failure, atherosclerosis, hypertension, and myocardial disease, which constitute early-onset CVD (3). The investigation of underlying mechanisms indicates that sterile or infection-induced inflammation triggers pro-inflammatory cytokines production and secretion via autocrine or paracrine pathways, which are major inducer of CVD onset (4). In most organisms, it is a crucial element for immunity to recognize aberrant DNAs via innate immune sensors. Recent studies have elucidated that double-stranded DNA (dsDNA) can be recognized by corresponding sensors, such as cyclic GMP-AMP synthase (cGAS), and then activates the innate immune system through mobilizing stimulator of interferon genes (STING) and downstream cascade signaling, which is one of the important signaling pathways to induce inflammatory response, and also represents an effective surveillance system against tissue damage and pathogen invasion. During this process, DNA from different sources will be trafficked into cytosol in different ways, including endocytosis, transmembrane diffusion, phagocytosis, and receptor-mediated endocytosis, thus activating the cGAS-STING signaling pathway (5). Emerging evidences implicate that cell-free DNA of human circulatory system is significantly correlated with CVD (6, 7), and multiple reports have also shown that DNA mediates the activation of cGAS-STING signaling, triggering primary cellular physiologies and tissues pathogenesis, such as CVD (8). Furthermore, the versatility of cGAS-STING has been uncovered in cellular physiological progress, plays a prominent role in disease pathogenesis. In this review, we will outline the cGAS-STING signaling pathway and DNA trafficking, summarizes the recent research advance on the effect of cGAS-STING signaling in varied cellular pathophysiological processes in CVDs.

## 2 cGAS-STING signaling pathway

**Figure 1:**
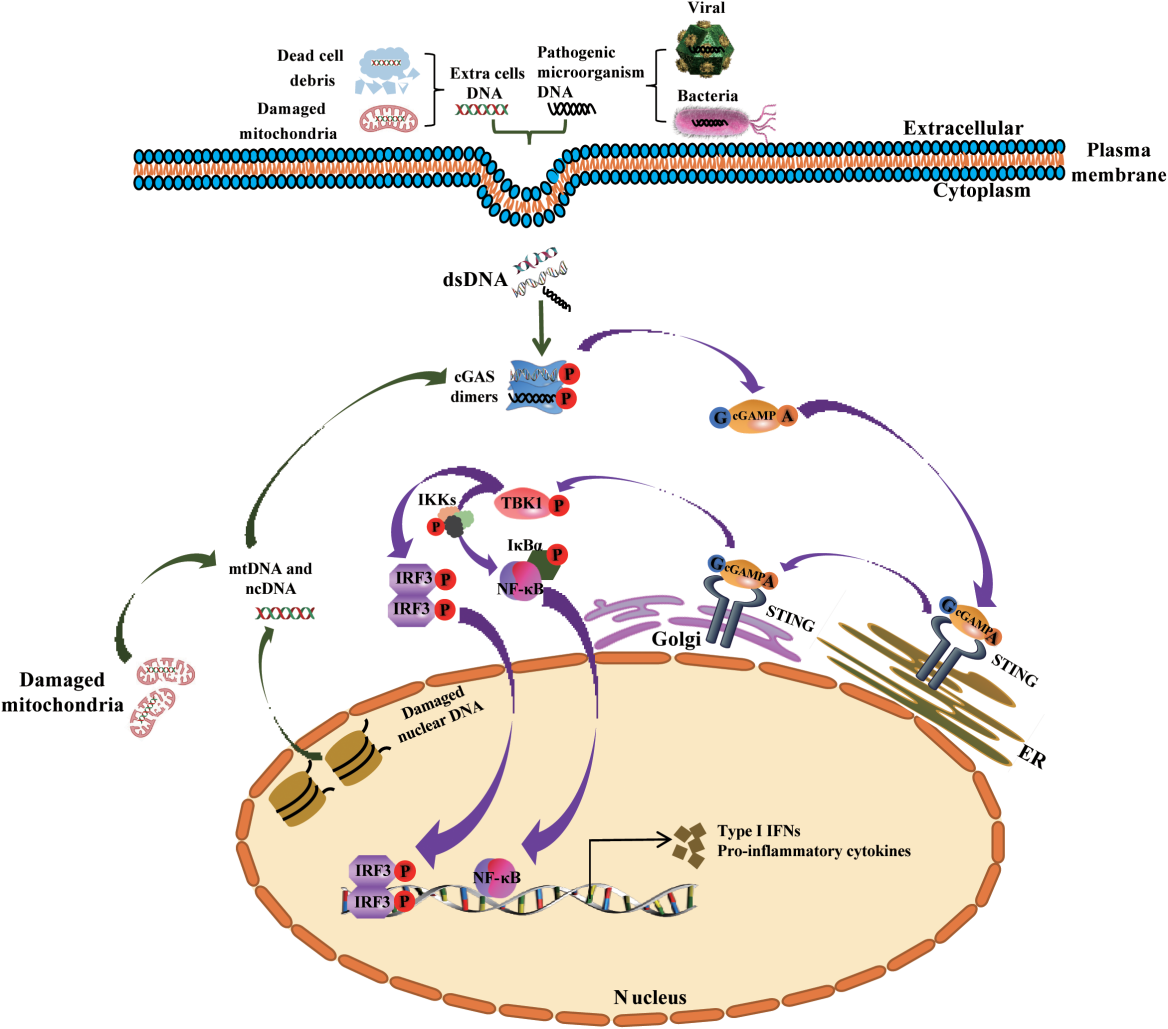
Mechanism underlying the activation of the cGAS-STING signaling pathway by cytosolic DNA, and the generation of type I IFNs and other cytokines. Cytoplasmic DNA from exogenous (bacterial and viral) and endogenous (damaged mitochondrial DNA or nuclear DNA or dead cells) sources bind and activate cGAS in the cytosol. Activated cGAS then uses ATP and GTP to synthesize cGAMP, the second messenger, which binds to the adaptor protein STING that is localized in the membrane of the ER. Following this, STING is trafficked to the Golgi apparatus, where it recruits and phosphorylates TBK1. Phosphorylated TBK1 phosphorylates IRF3 and the complex IKKs (comprising Ikkα, Ikkβ and Ikkγ) respectively, then phosphorylated IRF3 dimerizes and translocates into the nucleus to activates the transcription of IFNs, while Phosphorylated IKKs mediates IκBα phosphorylation and dissociation from NF-κB, triggers the translocation of NF-κB into the nucleus and promotes the transcription of pro-inflammatory cytokines. ncDNA, nuclear DNA; mtDNA, mitochondrial DNA; Golgi, Golgi apparatus; ER, endoplasmic reticulum.

Innate immunity is mammals’ first line of defense against pathogens and plays crucial roles in activating and orientating adaptive immunity. By recognizing various pathogens or damage-associated molecular patterns (PAMPs or DAMPs), pattern recognition receptors (PRRs) induce inflammatory response to trigger a signaling cascade, subsequently initiates autonomous cellular defense mechanisms to promote the production of soluble effectors, including interleukins (ILs), type I interferons (IFNs), and various other pro-inflammatory cytokines (9). Nucleic acids, originating from extracellular sources like viruses, bacteria, or dying cells or intracellular sources like damaged nuclei or mitochondria, serve as molecular patterns and are recognized by cytosolic nucleic acids sensors to activate downstream innate immune responses, which possesses important implications for monitoring infection, endogenous stress, or DNA damage (**Figure 1**). In 2006, it was first reported that IFNs were originally shown to be produced in mammalian cells in response to cytosolic dsDNA (10, 11). Up to date, more than five major sensors of dsDNA have been identified and described in mammals; these are toll-like receptor 9 (TLR9) (12), DNA-dependent activator of interferon (IFN) regulatory factors (DAI)/Z-DNA binding protein 1 (ZBP1) (13), absent in melanoma 2 (AIM2) (14), gamma-interferon-inducible protein-16 (IFI16) (15), and cyclic GMP-AMP synthase (cGAS) (16). These sensors all can initiate innate immune responses in CVD (17–20). Based on previous knowledge, cGAS functions as a pivotal cytosolic DNA sensor that interacts with STING to initiate the expression of type I IFNs and IFN-stimulated genes, this progress represents a canonical cGAS-STING signaling pathway.

Usually, cGAS can be stimulated and activated in the cytosol not only by intrinsic cellular self-DNA, including cytosolic micronuclei, cytosolic chromatin fragments, micronuclei DNA, and mitochondrial DNA (mtDNA) or DNA structures formed in the process of defective DNA replication, repair, and mitosis (21), but also by foreign DNA from viruses, microbial DNA, DNA from apoptotic cells, or extracellular vesicles (EVs) enclosing DNA from various sources, including but not limited to tumors (22). After activation by dsDNA, cGAS catalyzes adenosine and guanosine triphosphates (ATP and GTP) to synthesizes cyclic dinucleotide (CDN)-2’,3’-cyclic guanosine monophosphate-adenosine monophosphate (cGAMP). In this process, metal ions play a pivotal role for the activation of cGAS. After the cGAS combined with Mn^2+^, the sensitivity of cGAS to dsDNA will be significantly enhanced, and downstream cGAMP products also increased. In addition, Zn^2+^ also can coordinate the cGAS ribbon due to a unique zinc thumb of cGAS, which is critical for the interferon response, cGAS-DNA liquid-phase condensation, and subsequent cGAMP production (23). So, Zn^2+^ and Mn^2+^ ions dependency is an important characteristic of cGAS, which is critical for the generation of cGAMP, the activation of downstream signaling cascade and immune responses (23–25). Next, the generated cGAMP, as a second messenger molecule, activates STING by interacting with each other, and then activates downstream cascade signaling (16, 26).

cGAS is predominantly considered to be localized in the nucleus, cytoplasm, or on the plasma membrane, and efficiently recognize self-DNA and non-self DNA in a sequence-independent manner. However, it remains unclear how cGAS distinguishes self from non-self DNA. Barnett et al. suggested that, under physiological conditions, cGAS localization on the plasma membrane facilitates the recognition of foreign DNA. Because of this, when the mutants cGAS is incorrectly localized to the cytosol or nucleus, its recognition and response to the infected virus will be weakened (27, 28). Therefore, the precise localization of cGAS may be critical for its activity and ability to respond appropriately to both self- and non-self DNA. However, to date, the understanding of the detailed molecular regulatory mechanism is limited.

Increasing evidence shows that dsDNA mediated activation of cGAS-STING signaling is a vital mechanism in the development of chronic inflammatory diseases, involving in cancer, CVD, and metabolic diseases (8, 29). Based on the activation of cGAS-STING signaling induced by dsDNA from multiple sources (16, 30, 31), the mechanism underlying dsDNA release and trafficking is particularly important for understanding the detailed biological process of cGAS-STING signaling. The elucidating of this mechanism would help develop pharmacological agonists and antagonists that target cGAS-STING signaling in the treatment of relevant conditions.

## 3 Release and trafficking of cell-free DNA

**Figure 2:**
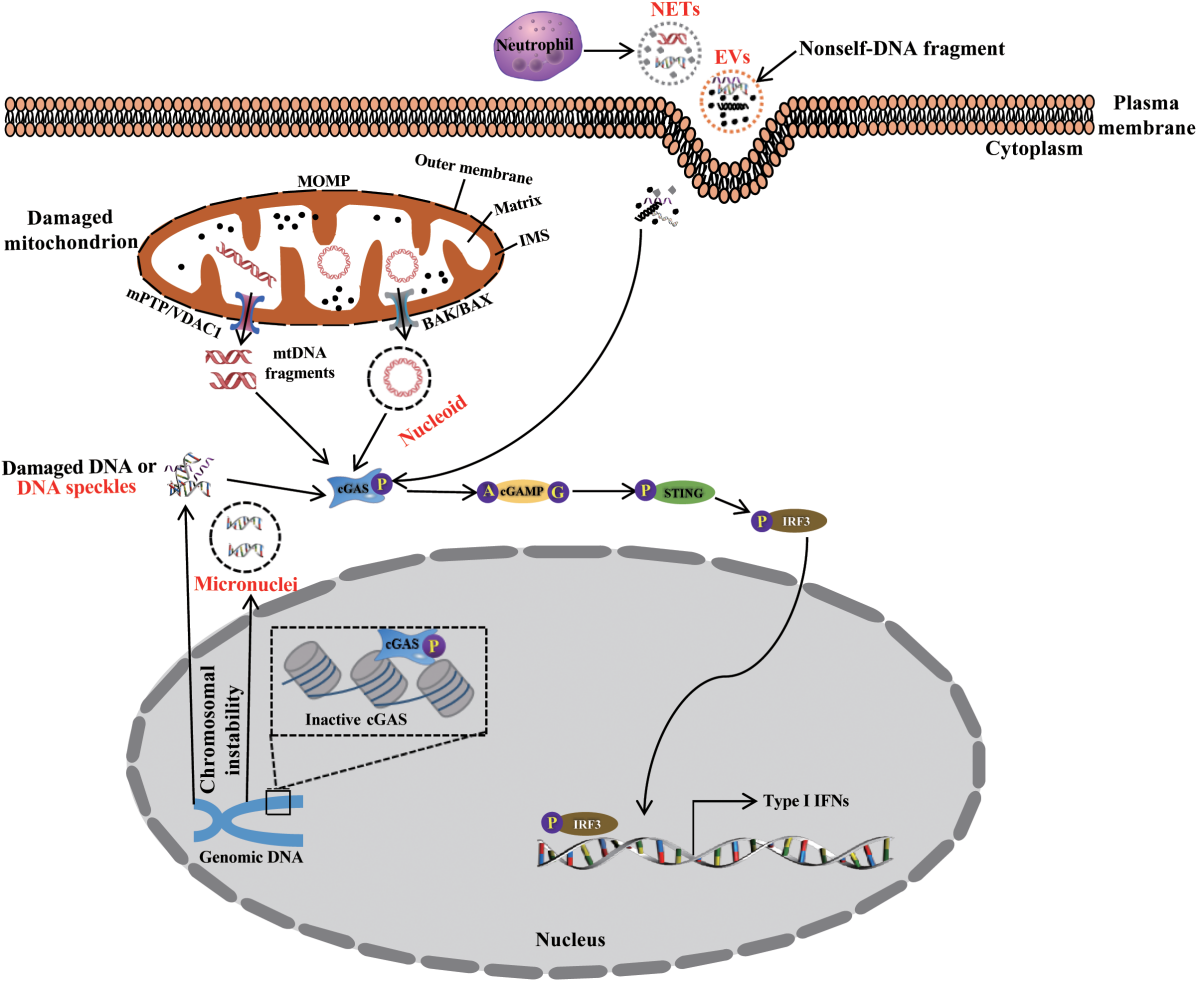
The packaging, release, and trafficking of DNA. During cGAS-STING signaling activation, DNA originating from endogenous organelle and exogenous organisms is packaged, released, and trafficked to the cytosol as nucleoids, micronuclei, EVs, NETs, speckles, and other complexes. These activate the cGAS-STING signaling pathway, thus priming immune response.

In 1940, the cell-free DNA (cf-DNA) in the human circulatory system was first identified by Mandel and Metais (32). Subsequent studies found that patients with rheumatoid arthritis or autoimmune disorders have higher amounts of cfDNA in their plasma and serum (33, 34). So far, cfDNA also have been found in a variety of liquid biopsy, including urinary (35), and saliva (36), amniotic fluid (35). According to different sources, circulating cfDNA is characterized as double- or single-stranded DNA fragments, mtDNA or chromosomal DNA, which are wrapped into different macromolecular complexes (**Figure 2**), such as mitochondrial nucleoids (37), nuclear micronuclei, EVs (38, 39), neutrophil extracellular traps (NETs) (40, 41), and speckles (42, 43), which originate from various cells through passive and positive DNA release mechanisms (44). However, the precise mechanisms remain unknown (45).

### 3.1 Mitochondrial nucleoids

The mitochondrion is a organelle with dynamic double-membrane and has its own genome (mtDNA) packaged into protein structures termed mitochondrial nucleoids. The human mtDNA contains 37 genes coding 2 rRNA, 22 tRNAs and 13 polypeptides respectively, these polypeptides are responsible for the assembly and function of mitochondrial respiratory complexes and play vital metabolic roles, such as the synthesis of ATP and reactive oxygen species (ROS), maintenance of calcium homeostasis, metabolite production, and energy metabolism (46). Mitochondrial dynamics and homeostasis maintenance play an important role in biogenesis, fission, fusion and mitophagy, which are essential for preserving the quality and function of the mitochondria, and are closely related to most metabolic disorders and CVD (46). Thereby, the control of mitochondrial dynamics and homeostasis may be considered as an effective therapeutic strategy (47). Recently, however, accumulating evidence suggests that mitochondria play a pivotal and direct role in the activation of immune responses by releasing mitochondrial metabolites, such as ATP (48, 49), succinate (50), cardiolipin (51), N-formyl peptides (52), mitochondrial transcription factor A (TFAM), and mtDNA (53) into the cytosol. These metabolites, due to the special chemical properties, can trigger innate immune response, and function as some of the most vital DAMPs (54, 55).

In general, pressure overload induces cells damage resulting in the leakage of DNA derived from mtDNA and (or) nucleus chromosome into the cytosol, which plays a pivotal role for the activation of cGAS-STING signaling, interferon-stimulating gene (ISG) expression, and eventually initiates an innate immune response, it is an important trigger for CVD onset (8). Based on that cfDNA has been identified in various fluid biopsy samples, such as plasma, serum, cerebrospinal fluid (CSF), and urine (56). The clinical studies suggest that sfDNA may be a potential biomarker of inflammation and a predictor of mortality due to high stability of cfDNA and the association of the concentration with the diseases (57). In plasma of healthy individuals, short-term elevated mtDNA molecules are rapidly cleared through phagocytosis, and then the level of circulating mtDNA return to normal physiological conditions (58). However, in pathological conditions, this is not the case. Chen et al. reported that high mtDNA content showed lower NK-cell frequency and higher IL-2 and TNF-a concentrations, and significantly poorer overall survival and progression-free survival in patients with gliomas after tumor resection, which may be attributed to the abnormal alteration of immune function, this result implied that leukocyte mtDNA content could act as an independent prognostic biomarker in patients with gliomas (59). Faizan et al. also found that cell-free mtDNA in the plasma of severely ill COVID-19 patients could know as a strong predictor of mortality due to COVID-19 triggered mtDNA release to activate cGAS-STING, and accelerate pro-inflammatory and pro-apoptotic progression (60). However, the regulation mechanisms on DNA released into circulating and trafficking into the target cells is still not clear.

Inside the cell, the release of mtDNA and the formation of specific structure are an important physiological and pathological regulatory processes. MtDNA is a circular intron-free structure containing unmethylated CpG motifs but lacking histone (61, 62). Because of insufficient safeguarding mechanisms, the mitochondrial genome is vulnerable to damage induced by ROS generated during respiratory and pathological metabolic events (63). Under normal physiological conditions, damaged mitochondria are removed through mitophagy. Nevertheless, interestingly, mtDNA cannot be cleared timely due to mitophagy dysfunction in most pathological conditions, such as CVD, which facilitates the accumulation of mtDNA in the cytosol (64). Now, a growing evidence indicates that mtDNA can be released into the cytosol via several ways related to mitochondrial, cellular, and local environmental stress. However, our understanding of the precise regulatory mechanisms involving mtDNA leakage into the cytosol is limited. TFAM is a regulator of the mtDNA replication and transcription, the expression is inhibited in the presence of mitochondrial stress induced by pressure overload or pathology, and then which promotes the release of mtDNA into the cytosol and activates cGAS-STING signaling, thereby aggravating pathophysiological conditions (37, 65). Typically, in the process of intrinsic apoptosis, the initiation of mitochondrial outer membrane permeabilization (MOMP) is a key process leading to caspase activation through the interactions of B cell lymphoma (BCL-2) family members (66). The interaction of BCL2 antagonist killer/BCL2-Associated X (BAK/BAX) triggers the formation of macropores on the mitochondrial outer membrane, and leading to apoptotic MOMP. Following this, mtDNA efflux occurs through the macropores into the cytosol and activates the cGAS-STING signaling (67, 68). This process is strictly regulated by the mitophagy proteins Parkin and PTEN-induced kinase 1 (PINK1) (69, 70). Kim et al. recently reported another mtDNA release mechanism involving the BAX/BAK-independent MOMP pathway (71). Under moderate oxidative stress, ROS induces the oligomerization of voltage-dependent anion channel 1 (VDAC1) on the outer mitochondrial membrane, promotes the release of mtDNA fragments, and activates the cGAS-STING signaling in the cytosol, leading to caspase activation or apoptosis. The process is regulated by YME1L, an efficient i-AAA protease required for *de novo* pyrimidine synthesis. The inhibition of YME1L expression results in the release of mtDNA into the cytosol via VDAC oligomers (72). Emerging evidence also shows that the opening of the mitochondrial permeability transition pore (mPTP) plays a crucial role in mtDNA leakage into the cytosol (73, 74). The RNA-binding protein transactive response DNA-binding protein of 43kDa (TDP43) normally localizes in the cytosol, nucleus, and mitochondria, is involved in the regulation of tRNA synthesis (75). After aggregation in the organelle, TDP43 becomes sensitive to oxidative stress. Following this, the opening of the mPTP causes mtDNA to be released into the cytosol, activates cGAS-STING signaling, which is a central regulatory mechanism in amyotrophic lateral sclerosis (ALS) (73). Tan et al. identified a novel regulatory mechanism attributed to SOD1 misfolding, which causes ALS and mitochondrial damage, and triggers the release of mtDNA and RNA : DNA hybrid formation in the cytosol in an mPTP-independent manner. This further activates the cGAS-STING signaling pathway and promotes ISGs expression (76). The above all evidenced demonstrate that the integrity of the mitochondrial membrane is critical for controlling mtDNA efflux and the activity of the cGAS-STING signaling under various conditions.

Two other mechanisms have also been proposed to explain the release of mtDNA in the extracellular space. In the first scenario, mtDNA is first released into the cytosol, subsequently enclosed in vesicles, which fuse with the cell membrane and eventually lead to mtDNA extrusion. An alternative mechanism involves the fusion of mitochondrial and cellular membranes, which triggers the release of mitochondrial contents (mtDNA) into the extracellular space (77). However, the precise regulatory mechanism underlying the release of mtDNA remains to be elucidated.

### 3.2 Nuclear micronuclei

The nucleus is also an important double-membrane organelle responsible for storing cellular genetic material. Nuclear DNA can enter the cytosol in various forms, including micronuclei, speckles, NETs, and genomic DNA damaged by nuclear envelope rupture initiated in response to improperly regulated mitosis and cell migration.

Nuclear DNA damage can be induced by various factors, such as DNA replication, chromosome instability, or other phenomena. Mitosis is a major contributor of them (78, 79). Damaged DNA “leaks” into the cytosol from the nucleus by forming a membrane-enclosed perinuclear package of DNA, termed micronuclei (80). The envelopes formed around micronuclei are easily broken due to its fragility, and subsequent damaged DNA is released into the cytosol, and activates cGAS-STING signaling pathway (80). To date, various regulatory mechanisms identified indicated that DNA structural damage occurs at different stages of the cell cycle or pathological process (81, 82). However, the precise regulatory mechanism on the form of micronuclei and release of damaged DNA is still unclear and remains to be further investigated.

### 3.3 NETosis

In response to various of exogenous stimuli (microbes and mitogens) or endogenous signaling activation (autoantibodies and aging), neutrophils produce NETs through a type of cell death known as “NETosis”, which occurs within chromatin surrounded by granular proteins, including neutrophil elastase (NE), cathelicidine LL37, and myeloperoxidase (MPO) (45, 46). There is increasing evidence that NETs are an important immune response factor that prevent the dissemination of pathogens by immobilizing and directly killing microbes (83). They also play an important role in certain autoimmune and other noninfectious diseases, including myocardial infarction (MI) (84), abdominal aortic aneurysms (85), and atherosclerosis (86). The induction of NETosis requires the synthesis of ROS via dependent or independent NADPH oxidase activity in neutrophils (87, 88). However, the exact regulatory mechanisms on the formation of NETosis remain unclear.

Emerging evidence suggests that dsDNA is one of the most important components of NETosis. Most dsDNA originates from nuclear DNA, but it has also been reported that mtDNA is wrapped in NETosis (89). There are two types of NETosis: suicidal NETosis and vital NETosis. The former is characterized by the rupture of the plasma and nuclear membranes, which results in the release of NETs from the cell after neutrophil activation; this process lasts for 5 to 8 h (90, 91). The latter does not involve neutrophils death and allows neutrophils to remain intact and functional. The process takes 5-60 min and is more rapid than suicidal NETosis (91). Interestingly, mtDNA, but not nuclear DNA, is involved in vital NETosis (92). The activation of both types of NETosis all depends on the presence of ROS in neutrophils (93). In addition, eosinophil extracellular traps (EETs) were also shown to recognize microbial pathogens, restrict their mobilization, and kill them in the extracellular space. The activation of this process depends on the presence of ROS, but the origin of DNA in this case is controversial (94, 95).

NETosis is an important factor for dsDNA transport and mediates dsDNA recognition as DAMPs by cGAS, finally activates the cGAS-STING signaling to trigger an immune response (41, 96). Mechanistically, when NETs are activated, nuclear DNA (chromatin) can be released from activated neutrophils in the form of NETs. Subsequently, cGAS recognizes the released dsDNA to activate the immune response, which is critical in sickle cell disease (96). Based on available data, in heart failure, NETs serve as a core component of an aseptic inflammatory response to strengthen myocardial tissue damage, such as fibrosis, ventricular remodeling (97). Ling and Xu found that in the onset of atrial fibrillation, myocarditis, or MI, neutrophils infiltrate the damaged tissue to exacerbate the inflammation response. Conversely, DAMPs may induce PRRs to promote NETs formation, but the precise crosstalk between NETs activation and the development of heart failure require further investigation (96).

### 3.4 Extracellular vesicles

EVs is first reported in platelet-derived procoagulant particles isolated from human plasma in 1946 (98). EVs are considered to be a heterogeneous group of cell-derived membrane-bound particles from various tissues, such as cartilage, tumor cells, or epithelial cells, released into body fluids (e.g., urine, cerebrospinal fluid, or serum). EVs play a core role in cell-to-cell communication, such as the exchange of proteins, lipids, or genetic material and involves in the regulation of multiple physiological and pathological processes (99). However, the exact formation and regulatory mechanism of EVs are the focus of research. Based on their various characteristics such as biogenesis, size, content, release pathways, and function, EVs are generally divided in four major populations: microvesicles (MVs), exosomes, apoptotic bodies, and retrovirus-like particles (100). Usually, EVs function as a special transporter of genomic DNA, mitochondrial DNA, or exogenous microbial DNA into the cytosol and activate cGAS-STING signaling (101, 102).

Dysfunction of apoptotic cells clearance initiates the formation of apoptosis-derived membrane vesicles (ADMVs), which is associated with autoimmune diseases such as SLE (103). ADMVs isolated from the sera of SLE patients contain dsDNA that activate the cGAS-STING signaling pathway, thereby inducing ISGs expression in THP-1 cells (104). It is an evolutionarily conserved regulatory mechanism, sensitive to microbial DNA, activates the host immune system to induce a defense response against microbial infection. Erttmann et al. reported that gut bacterial DNA is packaged into membrane vesicles, and then delivered into distal host cells, and subsequently activate the cGAS-STING-IFN-I axis but not toll-like receptor (TLR) signaling, or direct host-bacterial interactions to trigger host resistance to systemic viral infections (102). The interaction between T cells ant antigen-bearing dendritic cells (DCs) contributes T cell activation and initiates anti-pathogenic programs in DCs. When interaction signal is initiated, EVs containing genomic DNA and mtDNA are imparted the ability to trigger immune signals via the cGAS-STING cytosolic DNA sensing pathway and the expression of IRF3-dependent interferon regulatory genes in DCs (105). However, the formation and activation mechanisms of different EVs are yet to be elucidated.

### 3.5 Speckles

In addition to micronuclei containing condensed nuclear DNA, usually, during mitosis and DNA damage, damaged nuclear DNA is also released into the cytosol. Then, the DNA accumulated in the cytosol induces the formation of cytoplasmic DNA “speckles”, which, however, are less aggregated than micronuclei (42, 43, 82). Cytoplasmic DNA speckles mainly contain single-stranded DNA (ssDNA) and traces amounts of dsDNA (106). Mechanistically, the deficient endonuclease or exonuclease may be the key factor for DNA accumulation in the cytosol, chronic IFNs induction, and autoinflammatory phenotypes. Trex1 is a 3’-’5 DNA exonuclease localized in the cytosol, responsible for removing nucleotides from the nicked 3’ end of dsDNA. The knockout or mutation of *Trex1* causes the accumulation of damaged DNA in the cytosol, which contributes to the form of cytoplasmic speckles (107). MUS81 is an endonuclease participating in the conversion of nuclear DNA into cytoplasmic forms. In prostate cancer cells, MUS81 promotes the generation of cytoplasmic DNA, leading to type I IFN generation and the immune rejection of prostate tumors (108). However, in a recent study, MUS81-EME1 endonuclease complex negative was reported to regulate the spontaneous and HIV-1-mediated induction of type I IFN. In HIV-1 infection of cells, activation of associated MUS81-EME1 is to avoid accumulation of excess viral DNA by degrading viral nucleic acid, and subsequent preventing its sensing and type I IFN production, it is an effective way for HIV-1 to escape innate immune sensing (109). Although the ability of ssDNA to induce type I IFN expression is weaker than that of dsDNA because cGAS does not bind to ssDNA. Chowdhury et al. reported that ssDNA generated from damaged DNA forms double-stranded secondary structures, which may activate cGAS (107). In addition, ssDNA sequence with stem-loop-formed in HIV reverse transcription also exhibit high potency for cGAS activation (110). To sum up, several endonucleases or exonucleases are reportedly involved in the formation of cytoplasmic DNA speckles induced by DNA damage. However, limited information is available about the mechanisms underlying the accumulation of damaged DNA and the formation of cytoplasmic DNA speckles in the cytosol. Also, it is yet to be determined how cGAS-STING signaling is activated by ssDNA.

### 3.6 Others

Under certain circumstances, genomic DNA is also directly transported into the cytosol, and activates cGAS-STING signaling. Song et al. reported that ataxia-telangiectasia mutated (ATM) is responsible for DNA damage response, which contributes to reduce DNA damage, especially double-strand breaks, during the enhancement of DNA repair. Mutations in ATM results in the genomic DNA translocation into the cytosol and accumulation, thereby inducing cGAS-STING signaling activation in microglia as well as a variety of other cell types, while, inhibition of STING attenuates excessive cytokine production (111). Cell migration is the orchestrated movement of cells in multicellular organisms, often observed during embryonic development, wound healing, and immune responses. Usually, nuclear envelope rupture occurs frequently during cell migration and has potentially important roles in normal and pathological immune responses (112). Recently, several studies have reported that the migration of cells through narrow openings results in the transient rupture of the nuclear envelope. Once the nuclear envelope is not resealed rapidly with the assistance from the components of the endosomal sorting complexes required for transport III (ESCRT III) membrane-remodeling machinery, nuclear genomic DNA will leakage into the cytosol and activate cGAS-STING signaling pathway (113, 114). Retroelements are recognized for their pathogenic potential in response to the microbiota, such as allergies, autoimmunity, and inflammatory disorders. Evidence from the defined inflammatory mouse models (115) and human settings (116) demonstrates associates between retroelements and cGAS, and suggests that increased retroelement expression promotes aberrant accumulation of DNA, which may contribute to inflammatory responses through activating cGAS-STING signaling. Lima-Junior et al. found that enhanced endogenous retroviruses expression increases nucleic acids generation to promote inflammatory response caused by *S. epidermidis* in mice fed a high-fat diet via cGAS-STING, this result is in line with previous work demonstrated, including numerous pathologies, such as infection, autoimmunity, and cancer (117). It was also recently reported that TRIM18 and TRIM29, both of them are member of TRIM family proteins, function as a key negatively regulator for the innate immune response induced by viral infection (including DNA or RNA virus) through inhibiting the activation of cGAS-STING induced by accumulation of viral DNA or RNA in cytosol, and leads to the persistence of DNA or RNA viruses, however, knockdown or knockout of them separately enhances type I interferon production through activating of cGAS-STING induced by virus DNA or RNA, and subsequent to clear viruses. Mechanistically, TRIM29 induces K48-linked ubiquitination of STING, leading its rapid degradation, thereby triggers the persistence of Epstein-Barr virus (EBV) infections. While TRIM18 can stable protein phosphatase 1A (PPM1A) by inducing K63-linked ubiquitination to dephosphorylate TBK1, resulting inactivation of TBK1 to block cGAG-STING signaling, thereby dampening antiviral signaling to protect mice from viral myocarditis induced by coxsackievirus B3, viral pneumonia induced by influenza A virus PR8 strain and human adenovirus and herpes simplex encephalitis induced by herpes simplex virus type I. Base on the pivotal role of TRIM29 and TRIM18 in disrupting cGAS-STING axis, which may be a potential therapeutic target for CVD caused by persistent viral infection (118, 119). But, the release and trafficking of nucleic acid is a very complex and diverse process, and also an important prophase process for the activation of cGAS-STING signaling, involving a variety of pathophysiological process. So, the precise regulatory mechanism needs further investigation.

## 4 cGAS-STING signaling in CVD

**Figure 3:**
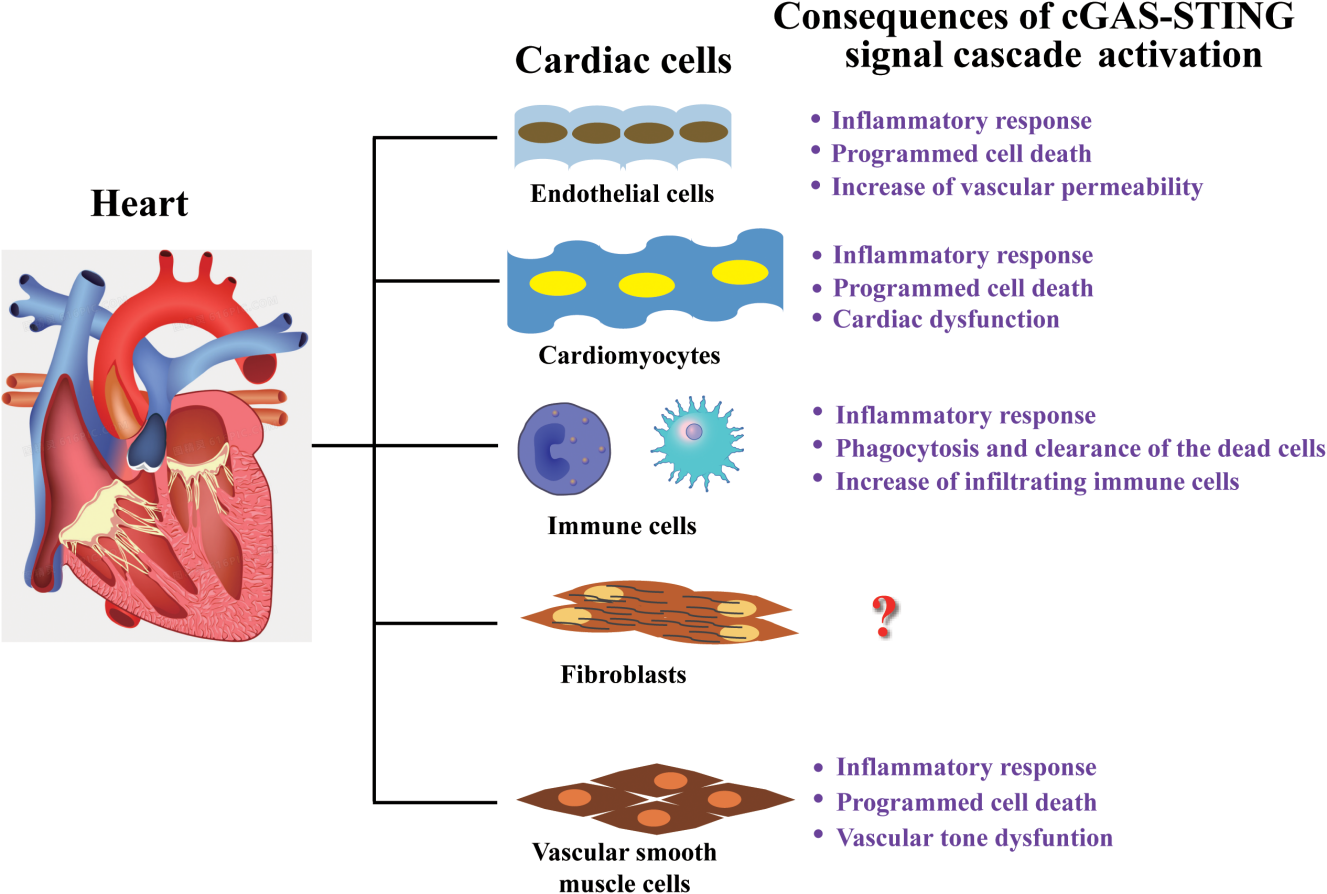
Cardiac cell-type compositions and the consequences of their response to cGAS-STING signal. Heart tissue comprises endothelial cells, cardiomyocytes, fibroblasts, vascular smooth muscle cells, and various types of immune cells. The consequences of cGAS-STING signaling cascade activation in various types of cells are mainly related to inflammatory response and dysfunction, but the response mechanism in cardiac fibroblasts have still not been reported and needs further investigation.

Multiple articles have reported a significant association between increased circulatory DNA (from microbial and host) and cardiovascular disease in humans (120, 121). Mechanically, cfDNA activates cGAS-STING response leading diverse cellular pathophysiological changes (autophagy, translation, metabolism homeostasis, senescence and cell death), subsequently induces pathological changes in the organ or tissues (heart failure, myocardial hypertrophy, inflammatory response) (**Table 1**). In turn, circulating cfDNA is also released from all major cardiovascular cell types, bacteria and virus, and trafficked in circulatory system by the form of EVs, mitochondrial nucleoids, nuclear micronuclei, NETosis and speckles, followly targeting to cardiovascular structures (137). Erttmann SF et al. reported that DNA-containing membrane vesicles from the gut microbiota delivering bacterial DNA into distal host cells, activate the cGAS-STING-IFN-I axis (102). For virus, DNA virus infection will cause its DNA fragments to be recognized by cGAS, which promotes activation of the cGAS-STING signaling, while RNA virus infection can also trigger cGAS-STING mediated innate immunity, but the effectors is mtDNA or chromosomal DNA in the cytosol induced by virus infection rather than RNA of virus origin (138). In addition, it has also been reported that virus proteins directly recognize and activate cGAS-STING (138). Therefore, various DNA released are important pathophysiological risk factors that contribute to the majority of cardiovascular damage caused by inflammatory responses, oxidative stress, programmed cell death, or excessive cell proliferation, eventually leading to heart failure, MI, and atherosclerosis (**Figure 3**) (8). Indeed, circulating cfDNA has been found in different body fluids both in healthy and non-healthy individuals and is identified as a crucial potential biomarker for CVD, with potential prognostic and diagnostic significance (139). However, there are also many problems, such as the standard concentration is uncertain, the detection method of circulating mtDNA concentration is different, and different teams have come to conflicting conclusions. So, according to the above statement, cfDNA as a biomarker has broad application prospects, but the detection and analysis methods need to be further standardized.

**Table 1:** The regulatory mechanism of cGAS-STING signaling pathway in experimental models of cardiovascular diseases.

Target tissue/cells	Diseases	Reason for the activation of cGAS-STING	The consequences of activation of cGAS-STING	Reference
Heart/Cardiomyocytes	Cardiac dysfunction affected by Alzheimer’s disease	Melatonin downregulation expression of cGAS to alleviate mtDNA mediated cGAS-STING signal activation	Cardiomyocytes apoptosis and cardiac dysfunction	(122)
Retinal microvascular endothelial cells	Inner endothelial blood-retinal barrier	Pathological stimuli (LPS or H2O2) induces mtDNA leakage into cytosol and activate cGAS-STING	RMEC inflammatory response and dysfunction	(123)
Heart/Cardiomyocytes	Diabetic cardiomyopathy	mtDNA leakage into cytosol induced by free fatty acids to activate cGAS-STING via NLRP3 inflammasome-dependent manner in cardiomyocytes	Cardiomyocytes pyroptosis and cardiac dysfunction	(124)
Endothelial cells	Impairment of endothelial angiogenesis and wound healing in diabetes	mtDNA leakage into cytosol induced by palmitic acid (PA) to activate cGAS-STING-IRF3, and then inactivate YAP	Inhibition of endothelial angiogenesis	(125)
Monocyte-derived macrophages	Clonal expansion of hematopoietic cells and higher risk of CVDs	Loss of function of DNMT3a- or TET2 resulted in impaired mtDNA integrity and activation of cGAS-STING	Type I interferon response	(126)
Mice vascular/Human aortic endothelial cells (HAECs)	Aging-related endothelial dysfunction	Aging or D-Galactose promotes mtDNA leakage into cytosol leading to activation of cGAS-STING	HAECs senescence and dysfunction	(127)
Endothelial cells	Obesity induced adipose tissue inflammation, insulin resistance and CVDs	Free fatty acids causes mitochondrial damage and leakage of mtDNA into the cytosol and activates cGAS-STING	Intercellular adhesion molecule 1 (ICAM-1) expression, monocyte-endothelial cell adhesion	(128)
Heart	Myocardial infarction	Interruption of IRF3-dependent signaling results in decreased cardiac expression of inflammatory cytokines and chemokines in the heart of post-MI mice	Reduction of inflammatory cell infiltration, attenuation of ventricular dilation and improvement of cardiac function	(129)
Vascular smooth muscle cells (VSMCs)	Chronic kidney disease	Oxidative stress induces mtDNA accumulation and activation of cGAS-STING in VSMCs	Triggering of the IFN-I response in VSMCs	(130)
Heart/Cardiomyocytes	High fat diet-induced cardiac anomalies	High-fat diet-induced cardiac anomalies, possibly through cGAS-STING	Akt2-AMPK double knockout accentuated high-fat diet-induced cardiac anomalies	(131)
Primary VSMCs	Vascular calcification	HDAC1-induced cGAS deacetylation results in its inactivation	cGAS is beneficial VSMCs calcification	(132)
Human cardiac endothelial cells	Radiation-induced inflammatory response	Radiation induces oxidative stress and mtDNA damage to activate cGAS-STING and inflammatory response in human cardiac endothelial cells	Activation of Inflammatory response leads to HCECest2 dysfunction, and followly the permeability of endothelial barrier caused by	(133)
Cardiomyocytes	Cisplatin cardiotoxicity	Cisplatin induces activation of cGAS-STING signaling pathway	Cardiomyocytes apoptosis	(134)
Cardiac myocytes	Hereditary dilated cardiomyopathy	LMNA mutation activated the cGAS-STING signaling pathway	The premature death in cardiac myocytes and DCM in mice	(135)
Human VSMCs	Vascular disease	Stress-induced premature senescence and stable expression of a telomeric repeat-binding factor 2 protein mutant (TRF2^T188A^) results VSMCs senescence, persistent telomere DNA damage and formation of micronuclei, which activates cGAS-STING-TBK1 signaling pathway	Persistent inflammation in vascular disease	(136)

### 4.1 cGAS-STING signaling in cardiomyocytes

As terminally differentiated cells, cardiomyocytes possess a limited regenerative ability. Therefore, the excessive damage and death of cardiomyocytes induced by pressure overload-related phenomena, including hypertrophy, apoptosis, necroptosis, pyroptosis, ferroptosis, and autophagy-mediated death, lead to the development of various cardiac diseases (140). In dilated hypertrophic hearts, such as diabetic cardiomyopathy and MI, the production of ROS induced by stress leads to mitochondrial damage, which triggers the release of mtDNA into the cytosol, activates cGAS-STING signaling, and results in the phosphorylation of IRF3 and NF-κB by the kinase TBK1 and IKKs respectively. Subsequently, phosphorylated IRF3 translocates into the nucleus from cytoplasm and increases NLRP3 expression and protein synthesis. Then, thioredoxin-interacting protein (TXNIP), an endogenous inhibitor of reactive oxygen scavenging protein thioredoxin, binds NLRP3 to form NLRP3/TXNIP complex, which recruits apoptosis-associated speck-like protein (ASC) and procaspase 1 to assemble the NLRP3 inflammasome. Thereby, the activation of NLRP3 inflammasome promotes the transformation of procaspase 1 into caspase 1, which subsequently converts pro-interleukin-1β and pro-interleukin 18 into mature IL-1β/IL-18, and simultaneously cleaves gasdermin D (GSDMD) into cleaved N-terminal of GSDMD (GSDMD-N), all of these contributes to the programmed cell death, hypertrophy and the release of pro-inflammatory cytokines in cardiomyocytes, and heart injury (124). The inhibition of STING signaling can considerably improve cardiomyocytes hypertrophy-induced cardiac fibrosis, macrophage infiltration, and inflammatory response in the heart of patients with diabetes and obesity (131, 141). In sepsis-induced cardiomyopathy, LPS activates STING-IRF3 signaling by stimulating cardiomyocytes, thereby activating the NLRP3 inflammasomes, ultimately resulting in inflammation, apoptosis, pyroptosis and cardiac dysfunction (142). These results all indicated the closely interplay between cGAS-STING axis and NLRP3 inflammasome. Further investigation also showed that STING, as an important signal molecular, promotes cardiac hypertrophy through regulating ER stress. Mechanistically, the deletion of STING not only attenuates expression of p-TBK1 and p-IRF3, but also alleviates the expression of p-PERK, p-IRE1α and p-eIF2α, so, in cardiac hypertrophy, STING could mediate ER stress through phosphorylating PERK and eIF2α (143). In addition, the effect was also investigated on cGAS-STING axis in cardiotoxicity involving TNFα induced the activation of NF-κB and AP-1 to promote inflammation and apoptosis. TNFα activates transcript factors NF-κB through phosphorylation of IKKs, which phosphorylates and degrades IκBα leading to the translocation of NF-κB into the nucleus, and promotes inflammatory cytokines production. Simultaneously, transcription factor AP-1, comprising c-Fos and c-Jun, can be phosphorylated induced by TNFα through the phosphorylation of JNK/MEK and translocated into the nucleus, subsequently phosphorylated AP-1 promotes transcription and activation of caspase 8 and caspase 3 cascade to initiates the apoptotic program. It is an important regulatory mechanisms for cardiomyocytes apoptosis induced by pressure overload (144, 145). Cisplatin, one of the cancer chemotherapeutic drugs, could activate the cGAS-STINGTBK1 to initiate IRF3-dependent innate immune response, resulting in the generation of IFNs and a NF-κB-dependent inflammatory response, and consequently which leads to synthesis of pro-inflammatory factors, such as TNFα. Then, TNFα promotes activation and translocation of transcription factor AP1 into nucleus to trigger caspase 3 dependent apoptosis signaling pathway to trigger cardiomyocyte apoptosis *in vivo* and *in vitro* (134). In the cardiac dysfunction of patients with Alzheimer’s disease and mice with APP1/PS1 mutations, APP1/PS1 mutations impair mitochondrial integrity leading to leakage and accumulation of mtDNA in the cytosol, which continuous stimulates cGAS-STING signaling resulting in reduction of cGAS-STING activity, and then reduced activation of autophagy and mitophagy via cGAS-STING signaling and further accumulation of damaged mitochondria and beta-amyloid in the cardiomyocytes, eventually initiating cardiac dysfunction (122). The probable explanation is that the prolonged stimulation of cytosolic mtDNA depletes cGAS-STING stores, but the exact mechanism is yet to be determined. To sum up, targeting cGAS-STING signaling in cardiomyocytes may be a promising therapeutic strategy for dilated or hypertrophic cardiomyopathy. However, the activation, cascade effect, and precise regulatory mechanism of cGAS-STING signaling are yet to be elucidated in cardiomyocytes and cardiac tissue.

### 4.2 cGAS-STING signaling in macrophages

Macrophages are the core regulator of immune system and the primary immune cells that reside in the heart tissue. Increasing evidence shows that macrophages mediating robust cardiac inflammatory responses that plays an important role in pathological cardiac remodeling and dysfunction initiated by pressure overload and ischemia. The cascade of inflammatory events includes an initial destructive phase, the generation of clearance of dead tissue, and a subsequent reparative phase involving myocardial healing (122, 129, 134, 142, 146, 147). In brief, heart injury induces the release of DAMPs from dead cells to activate macrophages resulting in the production of proinflammatory cytokines and chemokines in the initial inflammatory. Subsequent the expansion of macrophages populations contributes to the phagocytosis and clearance of the dead cells as well as the continuous release of cytokines and growth factors, which leads to the initiation of the healing process via the activation of myofibroblast proliferation and neovascularization of the injured myocardium (148). In the heart tissues of HF mice and patients, the inhibition of cGAS diminished inflammatory cell infiltration and inflammatory cytokine expression, and then promoted the transformation of macrophages to a reparative phenotype and alleviated inflammatory response, improved cardiac function, and fibrosis. Mechanistically, dsDNA was released from dead cardiomyocytes to stimulate the cGAS-STING-IRF3 signaling pathway in infiltrating macrophages, leading to a type I interferon response and inflammatory factor release, which then cause the apoptosis of cardiomyocytes and trans-differentiation of myofibroblasts (149). According to reports, transactive response DNA-binding protein-43kDa (TDP43) accelerates atherosclerosis progression by promoting lipid absorption and inflammation in macrophages. TDP43 promotes NF-κB activation contributing to inflammatory cytokine gene expression in macrophages by triggering mitochondrial DNA release, which activates cGAS-STING signaling-led inflammatory response. Additionally, TDP43 controls β-catenin expression and PPARγ complex formation to regulate CD36 scavenger receptor gene transcription and reinforce lipid uptake (150). It was obvious that cGAS-STING signaling, which mediates inflammatory responses in cardiac macrophages, plays a vital role in various cardiomyopathies. Thus, it is also highly promising as a therapeutic target. However, some regulatory mechanisms, such as the origin and release of dsDNA, require clarification.

### 4.3 cGAS-STING signaling in endothelial cells

By controlling the release of a spectrum of vasoactive molecules in vascular endothelial cells to regulate structural remodeling, inflammation, atherogenesis, and homeostasis of vascular (151). Meanwhile, there are other key factors that contribute to various vascular diseases, such as endothelial dysfunction, activation, inflammation, and barrier permeability (152). Recent studies also showed that STING over-expression results downstream signaling activation and vascular endothelial dysfunction, which is correlated with various cardiovascular diseases. This conclusion has also been confirmed in clinical and murine experiments. STING-associated vasculopathy with onset in infancy (SAVI) is an auto-inflammatory disease due to sutosomal dominant mutations in *TMEM173*, which encoding the STING genes. These mutants of STING reveal constitutive activation of STING and hypersensitivity to cGAMP, resulting in phosphoralation of TBK1 and IRF3 to promote interferon-response genes or other genes expression that mediates inflammation in vascular endothelial cells, which is an important cause to lesional skin from SAVI patients. Mechanically, the three mutations trigger the substitution of amino acid residues close to the STING dimerization site, and might result in the interfere with dimerization to form a stable dimer, which is important for the transduction of STING-IRF3 signaling (128). Metabolic stress triggers an increase of free fatty acid to initiate endothelial inflammation and dysfunction in diet-induced obesity mice. Mechanically, free fatty acid induces mitochondrial damage and release of mtDNA, and activates cGAS-STING-IRF3 signaling resulting inflammation and apoptosis. Generally, in patients with type II diabetes and SAVI, vascular endothelial dysfunction predisposes to microvascular and macrovascular complications (153, 154). However, STING functional inhibitory mutation exerts a protective effect against obesity-related cardiovascular disease in participants with an advanced age (155). It has also been reported that endothelial cells are the primary target in COVID-19. Thus, STING over-expression-induced endothelial dysfunction may be an important cause of thrombotic coagulopathy in patients with COVID-19, the possible explanation is that the metabolic stress of COVID-19 infection leads to the release of oxidized mtDNA, which promotes the activation of cGAS-STING-IFN cascade, but the precise mechanisms still need to be further clarified (156). In addition, various findings show that high levels of free fatty acids or a persistently high blood glucose is the essential link between obesity or type II diabetes and metabolic disorders, which impairs vascular mitochondria, leading to mtDNA release into the cytosol. This further leads to the engagement of cGAS-STING-IRF3 signaling to block angiogenesis, contributing to vascular complications induced by endothelial dysfunction. Mechanistically, phosphorylated IRF3 binds directly to the promoter regions of mammalian Ste20-like kinases 1 (MST1) and adhesion molecule-1 (ICAM-1) to promote MST1 and ICAM1 expression, which inactivates Hippo-YAP signaling. This represses cell survival, proliferation, and angiogenesis, and initiates an inflammatory response, leading to programmed cell death (125, 128). In all, the findings from these studies are sufficient to confirm that cGAS-STING signaling plays a central role in CVD. The release of mtDNA caused by different inducements is an important reason, except for STING mutant induced SAVI, however, the precise mechanistic and clinical studies on human CVD still need to be performed in the future.

### 4.4 cGAS-STING signaling in vascular smooth muscle cells

In blood vessels, VSMCs are the mail cell type with remarkable plasticity and localize in the tunica media of the vasculature. The most basic and important function of VSMCs is control systemic and local pressure through regulating vascular tone, e.g., the elastic rebound of the artery and extracellular matrix (ECM) generation (157, 158). Numerous findings suggest that VSMCs dysfunction is associated with several cardiovascular diseases, such as MI, hypertension, arterial aneurysms, and atherosclerosis. In this section, the mechanisms underlying the relation of VSMCs and intracellular cGAS-STING signaling in CVD will be summarized and discussed. In patients with insufficient development of coronary collateral artery, IFN-I signaling was strengthened. Whereas the inhibition of IFN-I signaling promoted arteriogenesis and VSMCs proliferation in mice (159). A fatal vascular disease, term sporadic aortic aneurysm and dissection (AAD), is characterized by persistent loss of aortic smooth muscle cell and the degradation of ECM. Interestingly, the release of cytosolic DNA and the significant activation of the STING signaling pathway are monitored in human sporadic AAD tissues. In mice with sporadic AAD, inhibition of STING significantly reduces aortic enlargement, dissection, and rupture in both the thoracic and abdominal aortic regions (160). Mechanistically, an integrated bioinformatics analysis reveals that STING contributes to multiple aortic smooth muscle cell response induced by stress, including ROS synthesis, DNA damage, inflammatory response, and cells death. ROS induces DNA damage and the release that activates the cGAS-STING-TBK1-IRF3 signaling axis and induces aortic smooth muscle cell death (apoptosis and necroptosis). Extracellular vesicles containing DNA may also contribute to the initiation of vascular disorders, such as atherosclerosis. Several studies have reported that microbial DNA is detected in human atherosclerotic plaques, which is related to monocyte recruitment and accumulation. Findings support that bacterial DNA also contributes to vascular dysfunction and inflammation mediated by the activation of cGAS-STING signaling. However, whether the consequence is attributed to the direct effect of microbial DNA on vascular and immune cells during atherogenesis remains elusive (161, 162).

### 4.5 The impact of cGAS-STING signaling on cardiac fibroblasts

Fibrosis is a common pathophysiological characteristic in a wide range of diseases, characterized by the excessive deposition of ECM components involving in collagens overgrowth, hardening or scarring of various tissues. In the heart, activated cardiac fibroblasts promote the synthesis and secretion of ECM components through proliferation and differentiation and are key effector cells in cardiac fibrosis. However, despite its global prevalence, effective therapies to inhibit or reverse cardiac fibrosis are still lacking, largely due to the complexity of the cell types and signaling pathways involved. The results based on clinical and murine experimental studies implicate that various stimuli from exogenous or endogenous sources, such as persistent infections, autoimmune reactions, allergic responses, chemical insults, radiation, and tissue injury, act as important inducers of chronic inflammatory response and lead to tissue fibrosis, including idiopathic pulmonary fibrosis, systemic sclerosis, and cardiovascular fibrosis (163). Recent reports suggest that transverse aortic constriction (TAC) induces inflammatory cell infiltration and inflammatory response through the cGAS-STING-IRF3-IFN signaling cascade to fuel pathological cardiac remodeling and fibrosis (146). Hu et al. also demonstrated that MI activates the cGAS-STING-IRF3 signaling of infiltrated macrophages to promote the fibrosis of cardiac fibroblasts, whereas a selective STING inhibitor significantly inhibits the cGAS-STING-IRF3 signaling, alleviates fibrosis and inflammation in mice, and alleviates the fibrosis of cardiac fibroblasts after co-cultured with the supernatants of bone marrow-derived macrophages (BMDMs) (164). These data reveals that cGAS-STING signaling regulates the inflammatory response in macrophages contributing to fibrogenesis of cardiac fibroblasts.

However, the effect of the cGAS-STING signaling on fibrogenesis in cardiac fibroblasts is rarely reported. So, further experimental investigations are needed to perform for elucidating the regulatory mechanisms of cGAS-STING signaling in cardiac fibroblasts, clarifying this phenomenon has important application value for the development of therapeutic drugs and clinical prevention and treatment of CVD.

## 5 Conclusion and perspectives

Globally, CVD remains the leading cause of human morbidity and mortality, and is also multifactorial chronic complex disease. In the past few decades, although some progress has been made in pathogenesis and prevention of CVD, the clinical treatment of CVD still has major limitations owing to poor prognoses. In recent years, inflammation (infection or non-infection-related inflammatory response) has been proposed as an independent risk factor for the development of end-organ comorbidities and the unfavorable prognosis for various chronic diseases, including CVD (164). In 2017, specifically targeted inflammatory therapy was first demonstrated to reduce cardiovascular events in high-risk subjects in a clinical trial (165). Mechanistically, various PAMPs and DAMPs activate the innate immune system through PRRs, triggering an inflammatory response that mediates local inflammation, metabolic disturbance, cell death, tissue injury, and cellular function impairment. However, the molecular mechanism by which inflammation contributes to CVD is yet to be fully elucidated.

As the research progresses, an increasing number of findings have implied that dsDNA is known to stimulate innate immune response. This present review highlights the ways of release and trafficking of dsDNA from different sources, and outlines one of the DNA sensors, emerging cGAS-STING signaling, and summarizes its essential role in CVD. Exogenous or endogenous dsDNA can be released and trafficked through mitochondrial nucleoids, nuclear micronuclei, NETosis, extracellular vesicles, and speckles to activate the cGAS-STING signaling pathway, phosphorylates IRF3, and triggers the synthesis of IFN. However, the regulatory mechanism underlying the formation and trafficking of different complexes containing dsDNA is yet to be further elucidated. This could provide a potential new drug therapeutic target for prevention and treatment of CVDs by improving dsDNA-mediated inflammatory response. In addition, dsDNA may also be directly translocated into the cytosol and activates the cGAS-STING signaling pathway, however, the detailed transport mechanism remains unclear.

Inflammatory responses induced by the activation of the cGAS-STING signaling can promote IFNs generation, which represents an effective therapeutic approach for solid tumors (166). However, in CVD, blood circulation is impaired owing to the damage and death of different cell types. To date, the regulatory role of cGAS-STING-mediated CVD has focused on immune cells-mediated systemic inflammation and endothelial and cardiomyocytes dysfunction and apoptosis. However, the roles of cGAS-STING signaling in several key cell types, such as cardiac fibroblasts and smooth muscle cells remain undetermined.

In conclusion, the activation of cGAS-STING signaling by dsDNA contributes to the initiation and development of CVD. However, the pathway may be more complex than mentioned in this review. Thus, better understanding of the release and trafficking of dsDNA upstream of cGAS-STING signaling is conducive to deciphering its role in the pathogenesis and development of CVD as well as the development of new targeted drugs.

## Author contributions

ZZ: Writing – original draft. CO-y: Validation, Writing – review & editing. QC: Validation, Writing – review & editing. ZR: Software, Writing – original draft. XG: Methodology, Writing – original draft. ML: Methodology, Writing – original draft. CL: Validation, Writing – review & editing. XY: Conceptualization, Writing – review & editing.
